# Epigenetic Responses to Anthropogenic Versus Natural Sources of Oil Exposure Differ in Wild Arctic Seabird Populations

**DOI:** 10.1111/eva.70125

**Published:** 2025-07-07

**Authors:** Wing‐Zheng Ho, Åsa Lind, Reyd Dupuis‐Smith, Frederic Dwyer‐Samuel, Samantha Pilgrim, George Gear, Rodd Laing, Gregg Tomy, Mark L. Mallory, Jamie Enook, Yasmeen Zahaby, Jennifer F. Provencher, Rowan D. H. Barrett

**Affiliations:** ^1^ Department of Biology McGill University Montreal Quebec Canada; ^2^ Biology Department Carleton University Ottawa Ontario Canada; ^3^ Nunatsiavut Government Nain Newfoundland and Labrador Canada; ^4^ The Centre for Oil and Gas Research and Development University of Manitoba Winnipeg Manitoba Canada; ^5^ Biology, Acadia University Wolfville Nova Scotia Canada; ^6^ Science and Technology Branch, Environment and Climate Change Canada Ottawa Ontario Canada

**Keywords:** Arctic, black guillemot, DNA methylation, epigenetics, oil pollution

## Abstract

Anthropogenic pollution can have detrimental effects on organismal physiology, behavior, and fitness, but the underlying genomic mechanisms mediating these effects are not well understood. Epigenetic regulation, such as DNA methylation, has been proposed as a potential mechanism mediating these effects, but currently, there are few studies in wild populations. Here, we examined the methylation patterns of liver tissues from black guillemot ( *Cepphus grylle* ) in regions of the Canadian Arctic with different histories of exposure to polycyclic aromatic compounds (PACs)—contaminants associated with hydrocarbons and petrochemicals. As compared to a reference site with minimal PAC exposure, the two sites with exposure to anthropogenic sources of PACs (shipping and spills) shared more differentially methylated regions (DMRs) than they did with the site experiencing chronic exposure to natural PACs (a hydrocarbon seep). Furthermore, we found that guillemots that have been exposed to anthropogenic PACs are characterized by having DMRs with significantly greater ratios of hypermethylated to hypomethylated DNA versus the population experiencing chronic exposure to natural PACs. However, birds from all three sites with elevated PAC exposure shared a core set of DMRs, implying that there are some consistent methylation responses to this family of compounds. Taken together, these results imply that the specific composition and exposure length of PACs can influence the direction of the epigenetic response. The identified DMRs serve as a genomic resource for further research investigating the functional role of DNA methylation in response to anthropogenic oil pollution.

## 1 Introduction

With decreases in the extent of Arctic sea ice due to climate change, anthropogenic activity has increased in Northern Canada, notably in the form of ship traffic, which has led to an increased risk of oil spill events (Mudryk et al. 2021). Oil contaminants such as polycyclic aromatic compounds (PACs) are a potent stressor that can cause widespread damage to local organisms, ranging from lethal effects via acute exposure to long‐term effects via sublethal chronic exposure. Prolonged exposure can lead to health, growth, or reproductive problems in affected individuals (Brette et al. 2014; Eppley and Rubega 1990; Fowler et al. 1995; Fry and Lowenstine 1985). These effects can have severe population consequences, as documented by the delayed recovery of harlequin duck ( *Histrionicus histrionicus* ; Iverson and Esler 2010) and sea otter ( *Enhydra lutris* ; Esler et al. 2018) populations for 14 and 20 years after oil spills, respectively. However, while the short‐term physiological impacts of oil spills have been well studied, the underlying molecular mechanisms mediating these effects are not as well understood—particularly in wild populations (but see Crump et al. 2016; Zahaby et al. 2021, which use transcriptomic approaches to monitor responses to contaminants in Arctic seabirds).

Among the hundreds of PACs, 16 compounds have been identified as priority contaminants by organizations such as the U.S. Environmental Protection Agency (USEPA), the International Agency for Research on Cancer (IARC), and the Canadian Council of Ministers of the Environment (CCME). These PACs have two or more aromatic rings, are non‐polar, stable, and hydrophobic, making them highly resistant to biodegradation and more likely to accumulate in soil or sediment (Adeola and Forbes 2021; Alaba et al. 2018; Lemaire et al. 2019; Sullivan et al. 2019). There are several other groupings of PACs that are often considered in toxicological studies. High molecular weight (HMW) PACs with four or more aromatic rings are more carcinogenic or mutagenic than low molecular weight (LMW) PACs with fewer aromatic rings (Agency (USEPA) 2000; Costa et al. 2017; Shi et al. 2018). However, in contrast to HMW PACs, LMW PACs tend to remain in solution and are readily available to marine organisms through ingestion or respiration, making them more toxic for marine biota, and are mostly associated with acute toxicity and genotoxicity rather than carcinogenic properties (Kieta et al. 2023). The solubility of LMW PACs also increases with temperature, making them more bioavailable in warmer seasons (National Research Council Canada Environmental 1983; Neff 1980). In general, the ratio of LMW:HMW PACs tends to be higher in anthropogenic sources of PACs (e.g., oil spills) than from natural sources (e.g., natural seeps; Provencher et al. 2020; Zahaby et al. 2025). These distinctions between LMW and HMW PACs highlight the importance of considering the specific characteristics of different PACs when assessing their potential impact on the environment. Despite this, we are not aware of any studies that have directly compared the functional consequences of exposure to distinct PAC types in wild populations outside of the laboratory.

The specific response of organisms to environmental stressors can also be influenced by their timescale of exposure. Long‐term, gradual exposure can often provide greater opportunities for adaptation and reduce the likelihood of extinction (Bell and Gonzalez 2009; Collins and De Meaux 2009). As such, slow rates of environmental change may allow for selection of beneficial mutations and changes in gene expression that contribute to adaptive responses in local populations (Bell 2013, 2017; Flores et al. 2013; Harmon and Pfennig 2021; Morgan et al. 2007; Samani and Bell 2016; Vanselow et al. 2022). In contrast, more rapid rates of environmental change can make acclimation or adaptation more difficult and lead to potentially detrimental phenotypic endpoints (Bay et al. 2017; Dolinoy et al. 2007; Turner 2009). As anthropogenic activities expose wild populations to increasingly variable and extreme changes in environmental conditions (Eyer et al. 2019; Hu et al. 2018; IPCC 2018; Stott 2016; Walther et al. 2002), it is crucial to understand the distinct mechanisms that might permit populations to respond to diverse perturbation scenarios, resulting from chronic to acute exposure.

Epigenetic mechanisms, including histone modification, ncRNA, and DNA methylation, can play a key role in rapid responses to environmental stressors (Dutta et al. 2018; Kilvitis et al. 2017; Lim et al. 2021; Verhoeven et al. 2016), and could potentially serve as a useful indicator of the impacts of oil contamination in marine wildlife. In particular, DNA methylation, which is the addition of a methyl group onto a cytosine and is usually associated with downregulation of gene expression, has been well studied and shown to be a reliable epigenetic marker as it is relatively stable, and not easily degraded during long‐term storage (Moore et al. 2013). With advances in DNA methylation sequencing methods such as whole genome bisulfite sequencing (WGBS), it is becoming feasible to investigate how pollutants can induce changes in DNA methylation in wild populations of animals (Chen et al. 2021; Hu et al. 2021; Laine et al. 2021; Zhang et al. 2021). Variation in DNA methylation may provide a mechanism to avoid declines in fitness when individuals are exposed to environmental changes (Janowitz Koch et al. 2016). These changes can also act on shorter timescales than genomic adaptation (Bossdorf et al. 2008) and may persist across generations (Head 2014). However, the relevance of epigenetic responses to varying timescales of hydrocarbon exposure in wild populations remain unclear. Additionally, we are not aware of studies that have directly compared the functional consequences of exposure to distinct PAC types on the epigenome of animals outside of the laboratory.

Research investigating DNA methylation changes induced by hydrocarbon exposure in wild animals has generally focused on global methylation responses rather than site‐specific methylation changes, with these global shifts showing inconsistent patterns across studies. For example, a study in juvenile red drum ( *Sciaenops ocellatus* ) found significant associations between high PAC exposure and hypomethylation in global methylation levels (Cañizares‐Martínez et al. 2022), whereas a study on double‐crested cormorants ( *Phalacrocorax auritus* ) found no significant association between airborne PAC exposure and global methylation levels (Wallace et al. 2018). Analyzing site‐specific methylation level differences can be useful because it enables the identification of differentially methylated regions (DMRs) that are associated with variation in ecologically relevant phenotypes and behaviors (Schrey et al. 2012). Site‐specific methylation data can help to identify differentially methylated CpG sites within specific gene regions, such as promoter regions or enhancers, which may be particularly important for regulating gene expression (Hu et al. 2014; Ko et al. 2013). Therefore, techniques such as WGBS can be valuable by permitting detection of CpG loci and the analysis of DNA methylation at single‐base resolution across the genome (Beck et al. 2022).

Here, we used WGBS to investigate whether exposure to natural and anthropogenic sourced PACs was significantly associated with DNA methylation patterns in populations of a wild seabird, the black guillemot ( *Cepphus grylle* ). The black guillemot is a valuable model species in ecological and environmental studies, given its broad distribution across North Atlantic and Arctic marine regions and its sensitivity to environmental changes (Piatt et al. 2007). The black guillemots that breed along the eastern shorelines of Baffin Island are thought to move potentially hundreds of kilometers away from their colony during winter, but the exact migratory patterns of this species remain largely unknown (Butler et al. 2020). During the breeding season, black guillemots gather in dense breeding colonies and generally forage within 15 km of these sites (usually much closer; see Mallory et al. 2019), making them highly amenable for monitoring and sample collection for contaminants research and allowing them to serve as useful indicators of oil exposure at the ecosystem level (e.g., Kuzyk et al. 2003; Piatt et al. 2007). The species' dietary reliance on benthic prey makes it vulnerable to marine pollutants as they dive to a depth range of 15–18 m (Shoji et al. 2015). Previous studies have observed guillemot population declines correlating with increased polychlorinated biphenyl (PCB) levels (Hoffman et al. 1996; Kuzyk et al. 2003). Thus, the black guillemot offers a versatile model for examining anthropogenic impacts on marine ecosystems and providing insights into pollutant effects and climate‐related changes.

Our objectives were twofold. First, we aimed to identify how sources of hydrocarbon pollution affected the methylation response of exposed seabirds across a range of sites experiencing (1) acute exposure to spill‐related, predominantly LMW PACs (SPILL), (2) chronic exposure to shipping‐related, predominantly LMW PACs (SHIP), and (3) chronic exposure through a natural seep of predominantly HMW PACs (SEEP). Second, we compared how the methylation response of seabirds differed when exposed to a sudden, acute oil pollution event (the spill site) versus a chronic and more gradual oil exposure due to anthropogenic activity (the shipping activity site). By identifying DMRs that are unique to these different scenarios, our research contributes novel understanding about the epigenetic response mechanisms used by wild populations of animals exposed to anthropogenic stressors.

## 2 Materials and Methods

### 2.1 Site Descriptions

**FIGURE 1:**
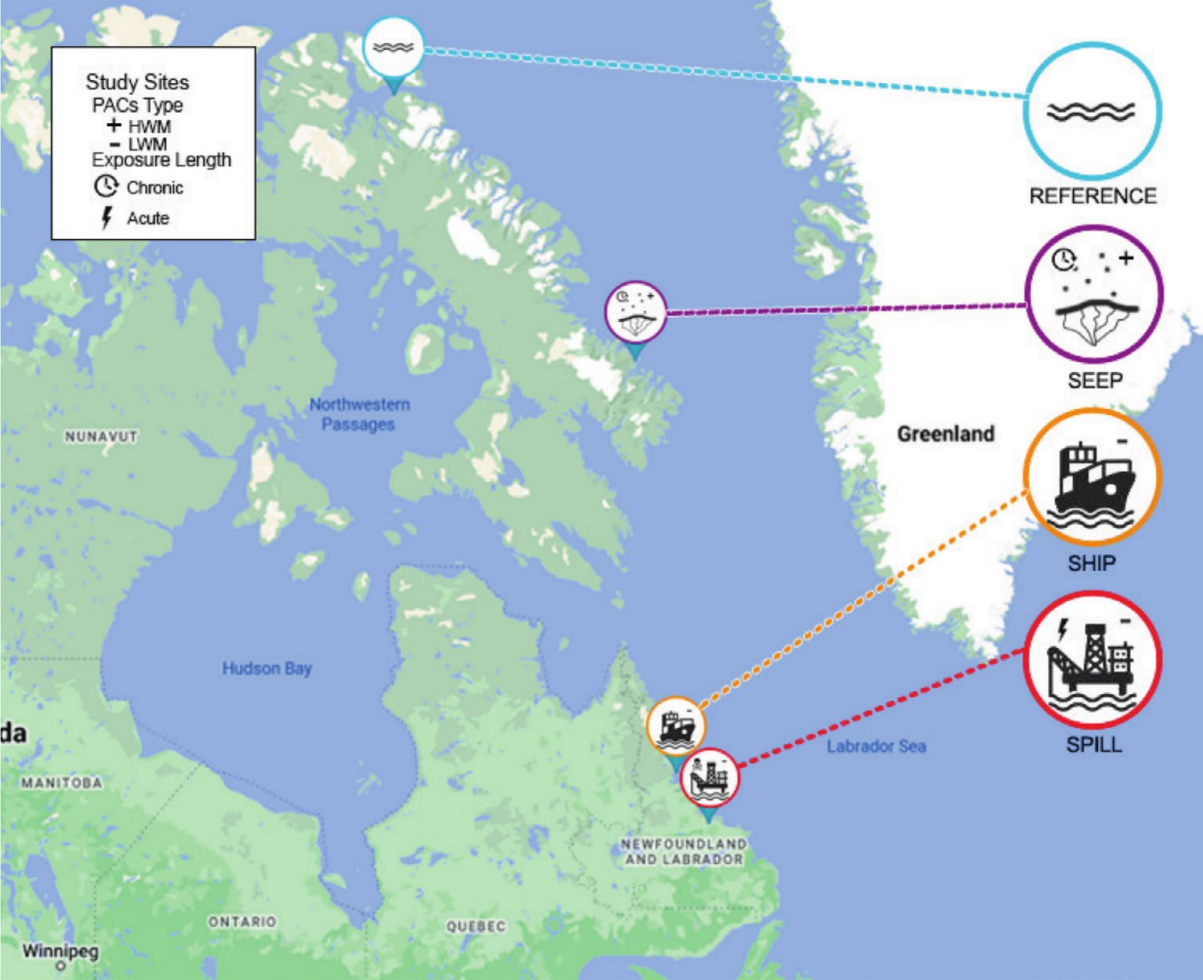
Locations of black guillemot colonies where liver samples were collected. Map data 2025 Google. Custom icons, annotations and legend were added to indicate study sites and exposure types in relation to PAC contamination.

**FIGURE 2:**
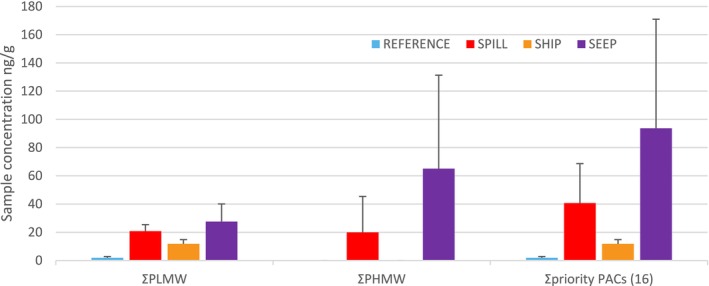
Polycyclic aromatic compounds (PACs) in liver tissues of black guillemot ( *Cepphus grylle* ) collected by Inuit hunters in Pond Inlet (REFERENCE), Postville (SPILL), Nain (SHIP), and Qikiqtarjuaq (SEEP) (using the most conservative limits of detections applied to datasets as published in Provencher et al. (2020)). Mean values are given with error bars representing the standard error (only the positive deviation values are shown for simplicity of the figure). The sum (Σ) of lower molecular weight (LMW) parent PACs (i.e., 2–3 ring compounds), and the sum of higher molecular weight (HMW) PACs (i.e., 4, 5 or 6 ring compounds), and the sum of the 16 USEPA priority PACs are presented.

**FIGURE 3:**
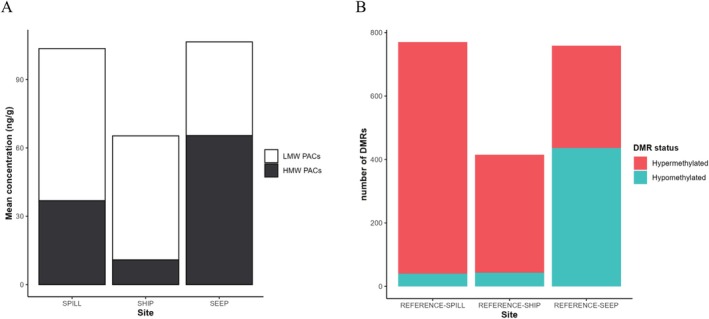
(A) Composition of PACs type LMW:HMW of the sites with significant exposure to PACs. (B) Distribution of hypo‐ & hypermethylated DMRs between each PAC‐exposed site and REFERENCE.

In June 2020, when black guillemots were breeding in the region, a spill of approximately 3000 L of crude oil occurred in Postville, Nunatsiavut (Nuka Research et al. 2023) (SPILL site). The high mobility and foraging behaviour of these birds (diving underwater to catch their prey) means that they are likely to encounter oil if it is present on the water's surface, sub‐surface, and in the benthic zone (Henkel et al. 2012; Wiese and Ryan 2003). In addition to the SPILL site at Postville, we leveraged three additional sites in Arctic Canada (one other in Nunatsiavut and two in Nunavut) for comparison in this study (Table 1; Figure 1). We selected Nain, Nunatsiavut (SHIP site) as a site that represented moderate shipping in the region because PAC exposure is elevated relative to the reference site due to higher levels of vessel traffic (Tables 1 and 2; Arctic Monitoring and Assessment Programme 2010; Harsem et al. 2011; Pizzolato et al. 2014). Like SPILL, this site is thus characterized by a higher ratio of LMW:HMW PACs (Zahaby et al. 2025; Figures 2 and 3A) but without the presence of an acute stress event such as an oil spill. In contrast, previous work has shown that birds at Qikiqtarjuaq, Nunavut (SEEP site), are exposed to natural hydrocarbon seeps, which are characterized by a lower ratio of LMW:HMW PACs (i.e., more HMW PACs) (Provencher et al. 2020; Figures 2 and 3A). Black guillemots at this site therefore experience chronic exposure to PACs with a different composition than those present at Postville and Nain in Nunatsiavut. Finally, Pond Inlet, Nunavut (REFERENCE site), was selected as a reference site for comparison due to a lack of natural oil seeps (Geological Survey of Canada 2022; van Luijk et al. 2020). Importantly, there is relatively low vessel traffic at REFERENCE and SEEP sites as extensive sea ice coverage in the summer limits the accessibility and navigability of vessels (Haas and Howell 2015; Howell and Brady 2019), which is not the case for the SPILL and SHIP sites (Tables 1 and 2).

**TABLE 1:** Characteristics of each study site.

Site	Site type	PAC type	Exposure type	Vessel based oil pollution	Natural seep oil
Pond Inlet, Nunavut	REFERENCE	N/A	N/A	Low vessel traffic (Pizzolato et al. 2014), no known acute commercial vessel oil spills in the region (Nunavut Impact Review Board 2019)	No known oil seeps near the breeding colonies (Bennett et al. 2014; Foster et al. 2015; Nunavut Impact Review Board 2019)
Qikiqtarjuaq, Nunavut	SEEP	HMW	Chronic	Low vessel traffic (Pizzolato et al. 2014), no known acute commercial vessel oil spills in the region (Nunavut Impact Review Board 2019)	Natural oil and gas seeps have been reported by Inuit harvesters and research vessels (Bennett et al. 2014; Foster et al. 2015; Nunavut Impact Review Board 2019)
Nain, Nunatsiavut	SHIP	LMW	Chronic	Moderate vessel traffic in the region (Arctic Monitoring and Assessment Programme 2010), no known acute commercial vessel oil spills in the region	No known oil seeps near the breeding colonies (Jauer and Budkewitsch 2010)
Postville, Nunatsiavut	SPILL	LMW	Acute	Moderate vessel traffic in the region (Arctic Monitoring and Assessment Programme 2010) documented acute spill of 3000 L of oil in the region in June 2020 (Environment and Climate Change Canada [ECCC], personal communication).	No known oil seeps near the breeding colonies (Jauer and Budkewitsch 2010).

**TABLE 2:** Polycyclic aromatic compound (PAC) concentrations (ng/g lipid weight) measured in liver tissue of black guillemots *(Cepphus grylle*) collected in the Pond Inlet (REFERENCE) region in 2022 by Inuit hunters. The sum (∑) of lower molecular weight (LMW) parent PACs (i.e., 2–3 ring compounds), alkylated LMW PACs (ALMW), and the sum of higher molecular weight (HMW) PACs (i.e., 4, 5 or 6 ring compounds), and their alkylated congeners (AHMW) are shown as well as parent heterocyclic (PHET) and alkylated heterocyclic compounds (AHET). The sum of the 16 USEPA priority PACs in wet weight (ww), and lipid weight (lw; ng/g) are presented. The mean, median, minimum (Min), maximum (Max) and standard deviation (SD) values are presented.

	Mean	Median	Min	Max	SD
Black guillemot—Pond Inlet (REFERENCE)					
ΣParent LMW	1.89	0.00	0.00	17.37	4.21
ΣAlkylated LMW	294.54	292.73	51.60	469.52	124.97
ΣParent HMW	0.00	0.00	0.00	0.00	0.00
ΣAlkylated HMW	80.98	63.38	18.64	266.47	59.92
ΣParent HET	0.00	0.00	0.00	0.00	0.00
ΣAlkylated HET	17.84	15.45	0.00	60.82	16.56
ΣUSEPA PAC lw	1.89	0.00	0.00	17.37	4.21
ΣUSEPA PAC ww	0.05	0.00	0.00	0.40	0.11
ΣAlkylated PAC	375.52	379.78	123.86	659.64	156.27
ΣPAC	506.39	393.89	180.34	1232.48	289.39

### 2.2 Tissue Choice

Liver tissue was selected for this study due to its critical role in detoxification and its relevance in assessing exposure to PACs. The liver has a large capacity for metabolizing xenobiotics, making it an ideal tissue for investigating short‐term exposure to contaminants in vertebrates (Custer et al. 2000; Hellou et al. 1996; Roscales et al. 2011). In birds, PACs have been detected in multiple tissues, including the liver, kidney, lung, brain, and muscle, but significant concentrations are often observed in the liver. For instance, Provencher et al. (2020) reported PAH concentrations ranging from 9.57 to 99.05 ng/g in the livers of marine birds.

### 2.3 Field Collections

We obtained fresh liver samples from bird carcasses of black guillemot, which were collected and flash frozen on‐site. All collections were conducted as approved under Canadian Council on Animal Care (CCAC) guidelines (Acadia University Permit ACC 02‐18) and federal, territorial, and scientific permits (ECCC NUN‐NWA‐18‐02, NUN‐SCI‐18‐02, GN‐WL‐2018‐004, NIRB‐17YN069, NPC‐148645, SC‐NR‐2021‐NU‐003, WL‐2021‐041, and NF‐NR‐2021‐NU‐002). The collected birds were of unknown sex, unbanded, and were not incubating eggs at the time of collection (Butler et al. 2020). Although we do not have age structure data from these populations, there are no significant levels of bycatch of black guillemots in these areas (Anholt et al. in press). Similarly, while this species is harvested across the range of this study, there is no evidence that it differs across the region, and thus, we do not think it is likely that there are systematic differences in the age structure of individuals sampled across sites. As described in detail in Provencher et al. (2020), teams of local Inuit hunters and researchers (all co‐authors) at each site collected the seabirds using 12‐gauge shotguns or 22 caliber rifles, while the birds were away from the breeding colonies and feeding on the water. Liver samples were collected from birds at the REFERENCE site (*n* = 21) in October 2022, the SPILL (*n* = 14) and SHIP (*n* = 16) sites in October 2020, and from the SEEP site (*n* = 28) in August 2018. Foraging distances of individuals are typically less than 10 km during our collection dates (Dehnhard et al. 2023), and thus, it is unlikely that birds frequented more than a single study site (minimum distance between sites is 225 km). Although there is a seasonal change in diet between Autumn and Winter, no significant changes have been documented between our collection dates in August and October (Baak et al. 2021).

### 2.4 PAC Profiles in Guillemots

PAC concentrations have been previously reported from the samples collected at the SPILL, SHIP, and SEEP sites (see Provencher et al. 2020; Zahaby et al. 2025). Here, we present for the first time PAC data from the samples collected at the Pond Inlet (REFERENCE) site, using the same procedures as those studies. The extraction of liver tissue and the analysis to determine the concentrations of 52 PACs and their alkylated congeners in each bird were done using the methods described in Idowu et al. (2018) and Provencher et al. (2020) at COGRAD in Winnipeg. To compare across the four sites, we examine groups of PACs and specifically the ratio between LMW and HMW PACs.

### 2.5 DNA Extraction and WGBS Library Preparation

Liver samples were stored in a −80°C freezer for a maximum of 1–2 weeks prior to extraction. DNA and RNA of samples of black guillemot were extracted using QIAGEN AllPrep DNA/RNA mini kit (Qiagen, Hilden, Germany) following the manufacturer's instructions. The DNA samples were submitted to the McGill Genome Center for whole‐genome bisulfite sequencing (WGBS) library preparation using the Illumina DNA Prep library kit. Samples were sequenced on three lanes of Illumina NovaSeq 6000 with 2 × 150 bp paired‐end reads at 15× target coverage.

### 2.6 WGBS Data Processing and Identification of Differentially Methylated Sites and Regions

We pre‐processed the raw sequencing data using the bioinformatics analysis pipeline methylseq v.2.3.0 through the workflow framework nf‐core (Ewels et al. 2020). We first performed quality checks on the raw sequencing reads using FastQC v.0.11.9 (Andrews et al. 2010), then trimmed the first two and last bases of every read using Trim Galore! v.0.6.7 (Krueger et al. 2021) after initial inspection of Bismark M‐bias plots. TrimGalore! was also used to remove base calls with a Phred score of 20 or lower, adapter sequences, and sequences shorter than 20 bases. We assessed the overdispersion factor (*λ*) using the mean and variance of methylation levels across the three sequencing lanes and found *λ* < 1, suggesting no evidence of overdispersion due to sequencing batch effects (Payne et al. 2017). We then mapped the processed reads to the black guillemot genome assembly (ASM1340106v1; NCBI BioProject PRJNA545868), deduplicated, and extracted the methylation call data using Bismark v.0.24.0 (Krueger and Andrews 2011) function *bismark_methylation_extractor*. On average, each sample yielded 1 × 10^8^ raw reads, and after quality filtering, we retained 1 × 10^8^ reads. Across all samples, on average, we found that 7 × 10^7^ (73.1%) of the quality‐filtered reads uniquely mapped against the black guillemot genome assembly. In total, we analyzed an average of 3 × 10^9^ cytosine bases, of which 2 × 10^9^ cytosines (67.08%) were methylated (Figure S6A,B). To test for population structure, we performed SNP calling using CgmapTools (Guo et al. 2018), starting with converting deduplicated mapped reads (.bams) to CGmap file format using the function *cgmaptools convert bam2cgmap*, and calculated pairwise weighted Fst between sites using VCFtools (Danecek et al. 2011).

We assessed the DNA methylation differences in each seabird population from the three exposed sites (SPILL, SHIP, and SEEP) relative to the REFERENCE site. Following this, the CpG loci were identified using the R package methylKit v.1.24.0 (Akalin et al. 2012) by importing the extracted methylation call data from Bismark via the function *methRead*. We then filtered the CpG loci for a minimum coverage threshold of 5 (lo.count = 5) and a maximum of 100 reads per base (hi.count = 100), excluded bases in the 99.9th percentile of coverage (high.perc = 99.9) using the *filterByCoverage* function, and normalized the filtered reads using the *normalizeCoverage* function to prevent any potential PCR bias. We used the function *unite*, with the parameter *destrand* set to TRUE, to merge all CpG loci such that they are covered in at least 80% of samples per group. These CpG loci were then used to calculate differential methylation via the function *calculateDiffMethDSS*, which is a beta‐binomial model from the DSS package (Feng et al. 2014) that calculates the differential methylation statistics using a beta‐binomial model with parameter shrinkage. We included sequencing lane as a covariate in the model to account for any potential batch effects, and further filtered by FDR < 0.05 to correct for multiple testing. We then used the *PCAsamples* function to perform a principal component analysis (PCA) on the methylation level of all CpG loci between each comparison pair, and between all three comparisons to identify general methylation patterns, and the *clusterSamples* function with ward.D agglomeration method to perform hierarchical clustering. Subsequently, differentially methylated regions (DMRs) were identified using the function *callDMR* in the R v.4.1.3 (R Core Team 2021) package DSS v.2.47.1 (Hao Feng 2019) with the default parameters: minimum length of 50 bp for DMRs, minimum 3 CpG sites for DMRs, and minimum percentage of CpG sites with significant *p*‐values (≤ 0.01) in DMRs at 50%, consistent with previous studies (Jeremias et al. 2018; Skjærven et al. 2018; Wang et al. 2021). We assessed the statistical significance of differences in the number of DMRs between study sites using a Pearson's Chi‐Square test via the function *prop. test* in R, and differences in the ratios of methylation status (hyper‐ vs. hypomethylated) using a Fisher's Exact Test. Furthermore, we also checked for any overlapping DMRs between each comparison pair, and between all three comparisons, using the genomic intersection tool Intervene v.0.6.5 (Khan and Mathelier 2017) and the *intervene venn* command. Statistical significance of the overlap between pairs of study sites of the reference site and each of all three sites, and the differences between the number of overlaps between comparisons were each calculated with a 10,000 round permutation test. All statistical tests were based on a heuristic that used the mean length of regions in our sets of DMRs (~400 bp) and the size of the genome (~1.3 × 10^9^ bp) to calculate the total number of possible DMRs (~3 × 10^6^ bp). All means are reported ± standard deviation unless otherwise noted.

### 2.7 Functional Analysis

We identified genes within the gene body region associated with shared DMRs via overlap with *bedtools intersect* (Quinlan and Hall 2010). We then used g:Profiler (Kolberg et al. 2023) to convert the gene symbol into ENSG ID to obtain functional information.

## 3 Results

### 3.1 PAC Profiles

The PAC concentrations from Qikiqtarjuaq (Provencher et al. 2020), Nain, and Postville (Zahaby et al. 2025) have previously been reported. Here, we focus on reporting new data on PAC concentrations from Pond Inlet (REFERENCE) and comparing PAC exposure between the four sites. We analyzed 52 different PACs compounds (Table S1). Method detection limits were all under 1 ng/g ww as reported in Idowu et al. (2018). Alkylated lower molecular weight (ALMW) PACs were detected at the highest concentrations (Table 2). Lower molecular weight parent PACs also contributed to total PAC burdens, indicative of a petrogenic PAC signature (Table 2).

Birds from each site showed evidence of distinct exposure histories to PACs, as reflected by clear differences in the concentrations of LMW and HMW PACs in their livers (Figures 2 and 3A). The mean concentration of the sum of 16 USEPA priority PACs for REFERENCE (1.89 ± 4.21 ng/g) was 21 times lower than SPILL (40.71 ± 104.43 ng/g), 6 times lower than SHIP (11.77 ± 12.00 ng/g), and 49 times lower than SEEP (93.67 ± 408.93 ng/g) (Figure 2). Among the sites with significant exposure to PACs, the ratio of LMW to HMW PACs differed significantly (Kruskal‐Wallis: *KW‐H*_3_ = 49.51, *p* < 0.0001). A Pairwise Wilcoxon test with Benjamini‐Hochberg correction indicated that SEEP had significantly lower LMW/HMW ratios than all other sites (0.63; *p* < 0.001). No significant difference was found between SHIP (1.81; *p* > 0.05) and SPILL (4.99; *p* > 0.05; Provencher et al. 2020; Zahaby et al. 2025; Figures 2 and 3A), showing that LMW PACs constituted the majority of the total PAC burden for birds at both SPILL and SHIP sites, while HMW PACs were in the majority at the SEEP site. Considering that PACs are typically rapidly metabolized by organisms (Shilla and Routh 2018), the detected concentrations most likely represent exposure to these compounds within a few days prior to the collection of the birds in their foraging zones around the breeding colonies.

### 3.2 Different Mechanisms of Response Between Sites With Different Exposure Histories

We found that the oil exposed populations varied in their number of differentially methylated regions relative to the reference population, with the sites exposed to the oil spill and chronic natural hydrocarbon seepage showing a greater number of DMRs than the site exposed to PACs via chronic shipping traffic. We identified 770 DMRs in the REFERENCE‐SPILL comparison, 435 DMRs in the REFERENCE‐SHIP comparison, and 759 DMRs in the REFERENCE‐SEEP comparison (Pearson's Chi‐Square test: REFERENCE‐SPILL vs. REFERENCE‐SHIP, *χ* ^2^ = 93.14, df = 1, *p* < 0.0001; REFERENCE‐SEEP vs. REFERENCE‐SHIP, *χ* ^2^ = 87.90, df = 1, *p* < 0.0001) (Figure 3B; Tables S2–S4). The direction of differential methylation varied according to the collection site of the birds, with higher ratios of hypermethylation in sites exposed to anthropogenic PACs compared to sites exposed to natural PACs (Fisher's Exact Test, *p* < 0.0001). In the REFERENCE‐SPILL comparison, we identified 5.19% of the 770 DMRs as being hypermethylated and 94.81% being hypomethylated. Similarly, in the REFERENCE‐SHIP comparison, we identified 9.89% of the 435 DMRs as being hypermethylated versus 90.11% hypomethylated. In contrast, in the REFERENCE‐SEEP comparison, we found that 57.44% of the 759 DMRs were hypermethylated, whereas 42.56% were hypomethylated (Figure 3B).

### 3.3 Shared and Distinct Patterns of Methylation Across Sites

**FIGURE 4:**
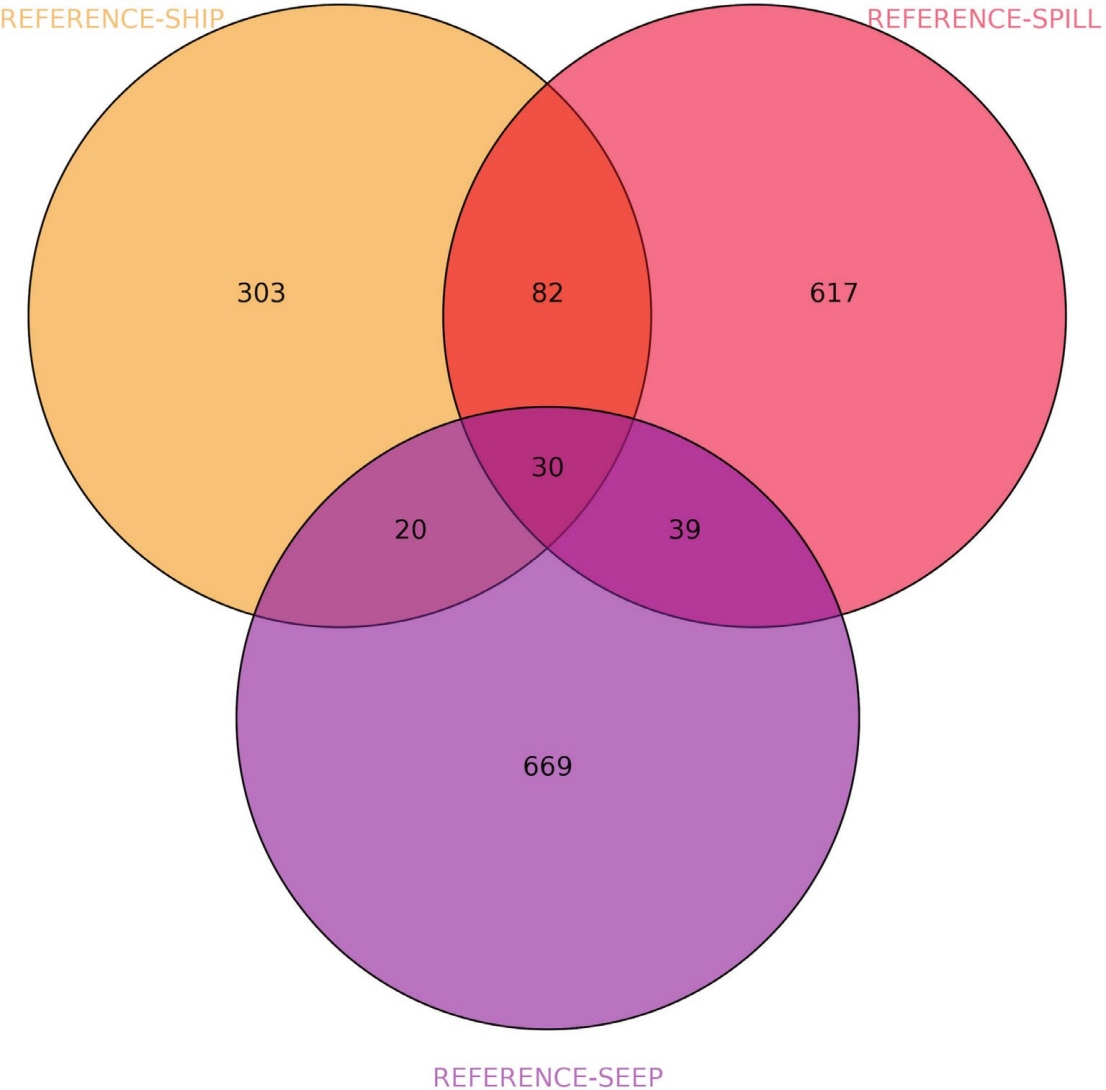
Venn diagram of DMRs shared between SPILL, SHIP and SEEP sites versus REFERENCE.

We found significantly more shared DMRs among sites than would be expected by chance, with a total of 112 (9.29%; Permutation test, *p* < 0.0001), 69 (4.51%; Permutation test, *p* < 0.0001) and 50 (4.19%; Permutation test, *p* < 0.0001) shared between REFERENCE‐SPILL and REFERENCE‐SHIP, between REFERENCE‐SPILL and REFERENCE‐SEEP, and between REFERENCE‐SHIP and REFERENCE‐SEEP, respectively (Figure 4; Table S5–S7). In total, 30 DMRs were shared between all three comparisons with the reference site (1.53%; Permutation test, *p* < 0.0001) (Table S8). However, the proportion of shared DMRs across sites was also associated with the type of PAC exposure, with more DMRs shared between the two sites exposed to anthropogenic PACs (SHIP and SPILL) versus the comparison of each of these sites to the site exposed to natural PACs (Permutation test, *p* < 0.0001).

We used principal component analysis (PCA) to explore the variation in methylation patterns between the different sites. The first two principal components (PCs) explained 16.79% of the variation in the REFERENCE‐SPILL comparison, 15.34% in the REFERENCE‐SHIP comparison, and 14.36% in the REFERENCE‐SEEP comparison when analyzing all CpG loci with 5× coverage across samples in the PCA (Figures S1A–S3A). When analyzing only DMRs in the PCA, the first two PCs explained 37.99%, 40.69%, and 34.70% of the variation in the REFERENCE‐SPILL, REFERENCE‐SHIP, and REFERENCE‐SEEP comparisons, respectively (Figures S1B–S3B). In both PCAs (using all CpG loci and using only DMRs), there was clear separation between the exposed sites and the reference site in all three comparisons. In a PCA analyzing all four sites together, the first two principal components (PCs) explained 10.77% of the variation for all CpG loci (Figure S4A) and 19.27% of the variation when using only DMRs. In the PCA analyzing DMRs only, there was some separation among the REFERENCE and SEEP sites versus the SHIP site and SPILL site (mainly on PC1) (Figure S4B).

### 3.4 Methylation Clustering Patterns and Genetic Variation Across Sites

Hierarchical clustering of all CpG loci showed SPILL and SHIP sites as largely grouping together, as did the REFERENCE and SEEP sites (Figure S5). The pairwise weighted Fst between sites ranges from 0.0003 to 0.005, indicating very low levels of genetic differentiation between populations. There is inconsistent correspondence between methylation clustering and patterns of genetic differentiation as well as geographic distance. SHIP and SPILL are located nearest each other in geographic space, group together in methylation space, and also show the lowest level of genetic differentiation (*F*st = 0.0003), whereas SHIP and SEEP show the highest level of genetic differentiation (*F*st = 0.005) but are not the farthest apart in geographic space and some individuals cluster close together in methylation space.

### 3.5 Functional Analysis

We identified 13 annotated genes (seven with ontology information and six without) that overlap with the DMRs shared between the REFERENCE‐SPILL and REFERENCE‐SHIP comparisons, six (four with ontology information, two without) with the DMRs shared between the REFERENCE‐SPILL and REFERENCE‐SEEP comparisons, and none with the DMRs shared between the REFERENCE‐SHIP and REFERENCE‐SEEP comparisons (Tables S12 and S13). Finally, six annotated genes (five with ontology information, one without) overlapped with DMRs shared between all three comparisons with the reference site (Table S14).

## 4 Discussion

Anthropogenic activities are leading to an increase in environmental stressors affecting wildlife health in Arctic populations, including PAC pollution (Hoffman et al. 1996; Mudryk et al. 2021). Epigenetic patterns may provide insights into the underlying genomic mechanisms mediating physiological responses to PACs as well as potentially serving as a useful indicator of the exposure history experienced by wild populations. We used a common and widespread seabird, the black guillemot, as a study system to test for differences in patterns of methylation among three sites, including an oil spill site, a high shipping traffic site, and a site with natural seepage of PACs exposed to varying types and exposure length of PACs to a reference site. In this methylome‐wide analysis, we found that the liver tissues of individuals exposed to different levels and types of PAC contamination possess methylome signatures with some shared characteristics but also distinctive aspects. Specifically, we identified a core set of DMRs that overlapped among all sites exposed to higher concentrations of LMW PACs than at the reference site, suggesting some consistent epigenetic mechanisms of response in black guillemots in relation to the PACs measured. Moreover, the number of DMRs shared between the two sites exposed to anthropogenic sources of PACs (shipping traffic and oil spill) were significantly higher than the number of DMRs shared between these sites and the site exposed to natural PACs. This observation was further corroborated by the results of the hierarchical clustering (Figure S5), which showed grouping of the two anthropogenic PAC‐exposed sites, separate from the cluster formed by the reference site and the site exposed to natural PACs. This suggests there are certain methylation responses that are likely to be specific to exposure to anthropogenic LMW PACs.

Further supporting the idea that black guillemots might be utilizing distinct epigenetic responses to natural versus anthropogenic sources of PACs were the significant differences in ratios of hyper versus hypomethylation between exposed sites and the reference. We found that DMRs at the SEEP site primarily reflected hypomethylation relative to the reference, whereas DMRs at the SPILL and SHIP sites overwhelmingly showed hypermethylation. The results from the SEEP site were consistent with previous studies that have reported hypomethylation patterns associated with exposure to HMW PACs (Quintanilla‐Mena et al. 2020; Shugart 1990; Teneng et al. 2011). It has been suggested that hypomethylation of gene promoters might allow increased expression of genes involved with stress response (Cavalli and Heard 2019; Metzger and Schulte 2016). In contrast, exposure of black guillemots to the mostly LMW PACs at SPILL and SHIP appears to have led to widespread DNA hypermethylation. However, these findings should be interpreted with caution. As the study was conducted on a limited number of wild populations, variability in DMRs among sites could also be influenced by a host of factors including, but not limited to, relatedness, age, sex, breeding status, migratory behaviors, diet, and individual metabolism of PACs (Caizergues et al. 2022; Chapelle and Silvestre 2022; Hu and Barrett 2017; Kilvitis et al. 2014; Laine et al. 2021; Yen et al. 2024; Zhang et al. 2021). However, the pairwise weighted Fst between sites was < 0.005 in all cases, indicating high levels of gene flow and suggesting that methylation variation across the sites is unlikely to be shaped by genetic differentiation between the populations. We were unable to collect sex data because we could not collect full intact carcasses for all birds, and black guillemots are sexually monomorphic, with sampling conducted after the breeding season in late August to October, so the birds were not incubating eggs. While we attempted sexing via PCR, we only had access to organ tissues rather than blood, and existing molecular methods that work well for blood (Griffiths et al. 1998; Kahn et al. 1998) were not successful with these tissue samples. Sex identification of this species using WGS/WGBS data is also not currently possible due to the lack of a chromosome‐level genome assembly. However, all birds were post‐hatch year adults, and the breeding status of the birds was consistent between sites. Furthermore, while data on diet differences among sites were not available, all sampled birds were in good condition, so food limitation is not likely to be a factor.

Exposure to other environmental stressors could also potentially impact the number of DMRs (Sepers et al. 2023). We are not aware of any differences in any of these factors among sites, but it is possible that the observed methylation patterns could be the result of a stress response to these factors (Baltazar‐Soares et al. 2024; Hu and Barrett 2017; Lämke and Bäurle 2017), rather than directly caused by PAC exposure history. Future studies could consider leveraging detailed knowledge of genetic and environmental variability among the populations and sites to help isolate the impacts of PACs from other factors. Furthermore, it remains unclear whether shared DMRs associated with different sites have a functional role in gene expression responses. In the future, studies leveraging both transcriptomic and methylome analyses will be important for uncovering these relationships.

Functional impacts associated with PAC exposure have been observed in previous studies in humans (Ünlü Endirlik et al. 2023), laboratory animals (Billiard et al. 2002), and wildlife species (Willett et al. 1997; Woo 2022), including birds (Perez‐Umphrey et al. 2018). For example, a study on sanderlings ( *Calidris alba* ; Bianchini et al. 2021) found that exposure to PACs reduced the expression of liver basic fatty acid binding protein 1 (Lbfabp) and hepatic lipase (Lipc), suggesting that exposure to PACs could hinder the processing of fatty acids, potentially leading to delayed migration departure timing (Bianchini and Morrissey 2018), which is vital for long‐distance migratory birds. Similarly, studies have shown that exposure to PACs can lead to the induction of specific genes, such as CYP1A (nestling herring gull *Larus argentatus* , Lee et al. 1985; chicken embryo, Lee et al. 1986; herring gull, Peakall et al. 1989), which plays an important role in PAC metabolism and serves as a recognized and widely used measure of both PAC exposure and the molecular effects of PACs (Jönsson et al. 2011; Lara‐Jacobo et al. 2019). Among the DMRs that we identified in this study which are shared across PAC‐affected sites, we identified 25 that overlap with annotated genes (Tables S12–S14). Among the subset of these genes that have ontology information available, two loci stand out as being likely candidates for having functionally important consequences due to their association with Circadian Clock pathways (Belinky et al. 2015). The protein encoded by the *SLC25A10* (Solute Carrier Family 25 Member 10) gene, which overlaps with DMRs shared between the SHIP and SPILL sites, can bind to the protein encoded by the *CLOCK* (Circadian Locomotor Output Cycles Kaput) gene. The *CLOCK* gene regulates the core circadian oscillator, influencing circadian and circannual rhythms (Bazzi et al. 2016; Panda et al. 2002), and has been linked to migration timing (Bazzi et al. 2017). Additionally, *CLOCK* has been shown to regulate *SLC25A10* to maintain glucose metabolism homeostasis (Cai et al. 2019), and suppression of *SLC25A10* represses de novo fatty acid synthesis (Mizuarai et al. 2005). The *Tgs1* (Trimethylguanosine Synthase 1) gene, overlapping with DMRs shared across all three PAC‐exposed sites, has been associated with reduced body weight gain and lipid accumulation when downregulated (Edwin et al. 2024). Thus, it is possible that changes to methylation due to PAC exposure could influence migration timing directly via the *SLC25A10* gene or indirectly via the *SLC25A10* and *Tgs1* genes by lengthening the time required for fueling because of reduced lipid accumulation and impaired glucose metabolism. We also performed GO term enrichment analysis but found no results due to the limited number of genes with ontology annotation. Greater understanding of the functional links between methylation changes and the expression levels of PAC‐associated genes is an important issue for further study and will hopefully be facilitated by the development of a chromosome level annotated reference genome in black guillemot.

The timescale of epigenetic responses to environmental perturbations in wild populations is poorly understood (Angers et al. 2020; Tian and Marsit 2018). In this study, we analyzed two sites, where wild black guillemots have experienced pollution from similar LMW PACs types but with different exposure histories. At SPILL, the population experienced an acute exposure of 3000 L of crude oil spilled into the water. In contrast, at SHIP, shipping traffic has been steadily increasing over the last 30 years with the opening of new shipping lanes (Oceans North Canada 2016; Pizzolato et al. 2014). While the general methylation difference relative to the reference site was remarkably similar between these two sites (97% and 97% of DMRs showing hypermethylation, respectively), we found 1.8 times as many DMRs at SPILL as at SHIP (878 vs. 539). This suggests that the acute PAC exposure caused by the oil spill might have led to a more widespread genomic response than that occurring at SHIP, where exposure to the 16 USEPA priority PACs was lower, and overall PAC exposure has been more gradual. Future temporal sampling will help to establish whether the broad genomic response we have observed at SPILL immediately following the oil spill might diminish to match SHIP more closely, or if the number of DMRs at SHIP will rise with further increases to shipping traffic and concomitant exposure to elevated PAC levels.

In this study, we explored the link between environmental oil exposure and methylation patterns in wild seabirds. We identified a core set of DMRs associated with oil exposure in black guillemot. In addition, we found evidence that varying composition of oil contaminants and length of exposure were associated with differences in methylation patterns. Development of a reference genome would allow for further functional analysis in future studies that could provide insights into the mechanisms underlying the observed epigenetic responses and help to disentangle the effects of oil pollution and other environmental stressors. Nonetheless, the loci identified here provide good candidates for further research investigating the functional role of DNA methylation in response to anthropogenic oil pollution.

## Conflicts of Interest

The authors declare no conflicts of interest.

## Supporting information


Data S1.

## Data Availability

Raw sequence reads are deposited in the SRA—BioProject ID: PRJNA1103249. Scripts and metadata are uploaded on GitHub: https://github.com/WingZHG/seabird.
